# Identification of rare genetic variants in Juvenile Idiopathic Arthritis using whole exome sequencing

**DOI:** 10.1186/1546-0096-13-S1-P144

**Published:** 2015-09-28

**Authors:** E Sanchez, S Grandemange, F Tran Mau-Them, P Louis-Plence, A Carbasse, E Jeziorski, M-C Picot, M Girard, TA Tran, B Isidor, S Poignant, S Tiriau, P Pillet, A-L Jurquet, I Touitou, D Geneviève

**Affiliations:** 1 Inserm U1183, IRMB, Montpellier, France; 2 Departement de gÃ©nÃ©tique medicale, Hopital Arnaud de Villeneuve, CHRU Montpellier, France; 3 Laboratoire des maladies rares et auto-inflammatoires, Hopital Arnaud de Villeneuve, CHRU Montpellier, France; 4 Departement de rhumatologie pÃ©diatrique, HÃ´pital Arnaud de Villeneuve, CHRU Montpellier, France; 5 Rhumatologie pediatrique, CHU Caremeau NÃ®mes, France; 6 Unite de genetique clinique, CHU Nantes, France; 7 Rhumatologie pediatrique, CHU Nantes, France; 8 Rhumatologie pediatrique, CHU Bordeaux, France; 9 Rhumatologie pediatrique, CHU Marseille, France

## Introduction

Juvenile Idiopathic Arthritis (JIA) is the most common form of chronic arthritis in children. JIA is characterized by onset of disease before the age of 16, with arthritis lasting >6 weeks, and with an unknown cause. Among JIA, seven sub-groups based on clinical and biological features have been individualized namely: systemic arthritis (sJIA) with autoinflammatory conditions, persistent and extended oligoarthritis (per-oJIA and ext-oJIA, respectively), rheumatoid factor-positive polyarthritis (RFpos-pJIA), enthesitis-related arthritis (ERA), psoriatic arthritis (PsA), and undifferentiated arthritis. Physiopathology of JIA is complex and JIA is considered to be a multifactorial disease due to the combination of genetic and environmental factors. Searching for genetic factors in JIA during the last decade, the introduction of genome-wide association studies (GWAS) and whole-exome sequencing have discovered several new loci associated with JIA susceptibility and have identified the disease-associated gene monogenic form of sJIA, respectively.

However, despite these novel knowledge, our understanding of JIA pathogenesis still remains poorly elusive and accumulating evidence supports genetic variability as playing a key role in JIA development.

## Objectives

The aim of our study is to identify novel genes involved in AJI in order to identify novel signaling pathways.

## Patients and methods

Thanks to a French National Hospital Research program (PHRC) including five french recruitment centers (Montpellier, Nîmes, Nantes, Marseille and Bordeaux), we have collected blood samples from 30 AJI patients and their unaffected parents. Based on clinical and biological data, the patients were classified in 5 groups: 1) oligoarticular form and negative autoantibodies (n=5), 2) oligoarticular form and positive antibodies (n=5), 3) polyarticular form and negative rheumatoid factor (n=10), 4) polyarticular form and positive rheumatoid factor (n=5) and 5) systemic form (n=5).

## Results

Using a whole exome sequencing trio strategy (i.e. patients and their unaffected parents) considering each family separately, we identified sequence variants in several candidate genes. Functional studies are ongoing in an attempt to demonstrate the pathological roles of the identified genetic variations.

## Conclusion

Our results could contribute to development of diagnostic tests, orientation of drug therapies as well as development of novel therapeutic targets. In addition, this study could demonstrate the ability to use whole exome sequencing in the context of the development of personalized medicine.

